# Strain-Sensing Properties of Multi-Walled Carbon Nanotube/Polydimethylsiloxane Composites with Different Aspect Ratio and Filler Contents

**DOI:** 10.3390/ma13112431

**Published:** 2020-05-26

**Authors:** Oh-Nyoung Hur, Ji-Hwan Ha, Sung-Hoon Park

**Affiliations:** Department of Mechanical Engineering, Soongsil University, 369 Sangdo-ro, Dongjak-Gu, Seoul 06978, Korea; ohnyung324@soongsil.ac.kr (O.-N.H.); jhwan618@ssu.ac.kr (J.-H.H.)

**Keywords:** carbon nanotube, aspect ratio, polymer composite, strain sensor, hysteresis

## Abstract

For filler composite systems used in strain sensor applications, piezoresistive effect, strain hysteresis, and repeatability are critical factors, which have to be clearly evaluated and understood. To investigate the effects of the aspect ratio and content of a multi-walled carbon nanotube (MWCNT) on the strain sensor properties of the composite, MWCNT/Polydimethylsiloxane (PDMS) composites with varying filler contents and aspect ratios were fabricated. In order to uniformly disperse MWCNTs on the polymer matrix, we used a three-roll milling method to generate high shear force for de-bundling MWCNTs. Mechanical and electrical properties of the MWCNT composites were evaluated for each case. In addition, through the cyclic stretching test, we optimized the strain-sensing properties of the MWCNT composites by considering their piezoresistive effects and strain hysteresis.

## 1. Introduction

Currently, various studies on pressure sensors and strain sensors are in progress [1,2,3,4]. Most commercially available strain sensors are metal-based and have a disadvantage in that their strain-sensing range is very limited. To improve the strain-sensing range, many studies have been aimed at developing a strain sensor with a wide strain-sensing range by adding various carbon-based fillers such as carbon black, carbon fiber, MWCNT, and graphene to the polymer [5,6,7,8,9,10,11]. MWCNT, one of the diverse fillers, is frequently used as a conductive filler because of its large aspect ratio. Due to the large aspect ratio of MWCNT, sufficient electrical paths are generated inside the composites even with the use of a small amount of MWCNT [12,13,14].

The piezoresistive effect is a key contributor to the sensing mechanism of the strain sensor. Piezoresistive effect describes a change in electrical resistance as a result of mechanical deformation [15]. Previous studies have been conducted to explore the factors influencing piezoresistive effect. From the results of those studies, piezoresistive effect is influenced by the dimensions and contents of the filler and the structure of the composite [3,6,16,17,18]. Some studies observed that electrical resistance did not return to the initial state even after the removal of the tension causing it from the composites [10,19]. This phenomenon is called hysteresis. In detail, hysteresis is a phenomenon in which a physical quantity is not determined only by the physical conditions at that time but also by the change process of the state that the substance has been in before. Former studies have shown that hysteresis is also affected by the dimension and content of fillers and the morphology of composites [9,20]. 

As seen above, the morphology of the composite is an important factor in determining both the piezoresistive effect and hysteresis; therefore, it is important to uniformly disperse conductive fillers on the composite. Ultra-sonication is a general method for dispersing MWCNTs on composites [16,21]. The method shows excellent dispersing effectiveness but poses a challenge with the dispersion of fillers on high viscosity composites. A solvent may be used to disperse fillers on high viscosity composites, but it causes inconvenience in terms of solvent removal during composite fabrication. Another method for conductive filler dispersion in high-viscosity composites is the three-roll mill method. This is a method of filler dispersion on composites that uses the shear force generated by three-rolls [22].

**Figure 1:**
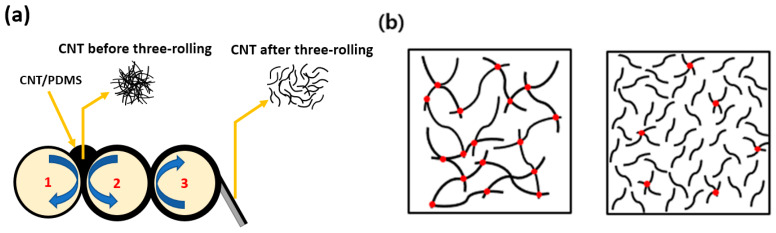
A scheme of (**a**) a carbon nanotube (CNT) dispersion mechanism during three-roll milling. (**b**) Morphology of long CNT composite (left) and short CNT composite (right) with the same filler content. Red dots indicate the electrical contact points.

In this study, we fabricated 0.07, 0.5, 1, 3, and 5 wt% MWCNT polymer composites using short MWCNTs and 0.4, 0.5, 1, 3, and 5 wt% composites using long MWCNTs to determine the effects of the aspect ratios and content of MWCNTs on the mechanical and electrical properties of composites during strain sensing. We used the three-roll milling method to attain even dispersion at high viscosity. Figure 1 schematically presents the mechanism of the three-roll milling method and the morphology of two types of MWCNT composites. In order to calculate the aspect ratios of the two different conductive fillers, their lengths were measured by scanning electric microscopy (SEM). In addition, the internal morphologies of the composites were also observed via SEM. Mechanical and electrical properties of MWCNT composites were measured by tensile and cyclic experiments, respectively. 

## 2. Materials and Methods 

### 2.1. Materials

Two types of MWCNTs were used as conductive fillers. The long MWCNT was purchased from JEIO (Incheon, Korea) and had a mean diameter of 5 nm, bundle length of 50–150 μm, and purity of >97.5 wt%. The short MWCNT was purchased from KB-Element Co., Ltd (Gyeonggi-do, Korea) and had a mean diameter of 5 nm, a length of 10–20 μm, and purity of >98 wt%. PDMS (Dow Corning, Sylgard 184, Midland, MI, USA) was adopted as the base polymer.

### 2.2. Fabrication of MWCNT/PDMS Composite

The paste was fabricated using a paste mixer (Daehwa, Seoul, Korea) and three-roll milling (Intech, Gyeonggi-do, Korea) for even dispersion of MWCNT on PDMS. The specific process was as follows: PDMS was first prepared with premixing elastomer(A) and curing agent(B) (mass ratio A:B=10:1), and then MWCNT was added to the PDMS. A paste mixer was then used to blend the paste at 500 revolutions per minute (rpm) for 30 s. Next, the paste was continuously mixed at 1500 rpm for 60 s. The MWCNTs were then uniformly dispersed on the composites by three-roll milling for 5 min. To measure the mechanical and electrical properties of the MWCNT/PDMS composites, a thin film was then made. The paste was further pressed and cured by a hot-press heating plate (Qmesys Inc., Gyeonggi-do, Korea), operated at 150 °C and 15 MPa for 1 h to obtain a thin and flat film with a thickness of 1 mm. 

### 2.3. Characterization

To measure the length of the MWCNT, small quantities of two kinds of MWCNTs were separately dispersed in chloroform (Daejung, Seoul, Korea) by ultra-sonication at a pulse of 20 kHz for 25 min. Next, the suspension was spin-coated on a silicon wafer in a three-step process. The first step was conducted at 500 rpm for 5 s. The second step was conducted at 2000 rpm for 30 s, while the third step was conducted at 500 rpm for 5 s. The spin-coated wafers were then dried at an ambient temperature for a day. The dried spin-coated wafers were then observed by SEM (Gemini SEM 300, ZEISS Inc., Land Baden-Württemberg, Germany). The equipment was operated at an accelerating voltage of 5 kV. To understand the morphology of MWCNT/PDMS composites, the composites were fractured in liquid nitrogen. The cross-section of the fractured composites was coated with Pt and then observed using SEM at a 5 kV accelerating voltage.

The mechanical properties of the composites were measured using a universal testing machine (UTM) (DRTECH Inc., Gyeonggi-do, Korea). Mechanical test samples of 1 and 5 wt% MWCNT/PDMS composites with a thickness of 1 mm were made according to ASTMD 638-5. All mechanical tests were performed at a cross-head speed of 50 mm/min and with a load-cell of 50 kgf (490 N), and five specimens were tested.

To measure the electrical properties and percolation thresholds of the composites, various content composites were made. For each content composite, five samples were made, and the average values of the electrical properties and percolation thresholds were obtained. The sample size was 50 × 5 × 1 mm^3^. The samples were ultraviolet (UV) etched for 300 s by a UV ozone room (JSE Co., Seoul, Korea) to enhance the electrical contact between the samples and the silver paste (Protavic, Levallois-Perret, France). The silver paste was then covered on both ends of the surface of the samples as electrodes. After this process, the samples were cured in a stove at 120 °C for 1 h. After that, their resistance was determined using a two-wire method (Keithley DMM 7510 multi-meter, Keithley, Cleveland, OH, USA).

The hysteresis property tests were conducted using a 3-dimensional stretching machine (NAMIL optical instruments Co., Incheon, Korea) and a two-wire method. The samples were stretched from steady state to 30% strain and then released back to steady state at the same speed; the changes in their resistances were measure simultaneously. The released state was maintained for 1 min. The entire process was repeated over 30 cycles. The piezoresistive property tests were conducted immediately after the hysteresis property tests. The samples were stretched from steady state to 30% strain and then released to steady state continuously over 20 cycles without a rest time. During the stretching and releasing cycles, the resistances were measured in the same way as the resistance measurements conducted for the hysteresis tests.

## 3. Results and Discussion

### 3.1. Morphological Analysis

**Figure 2:**
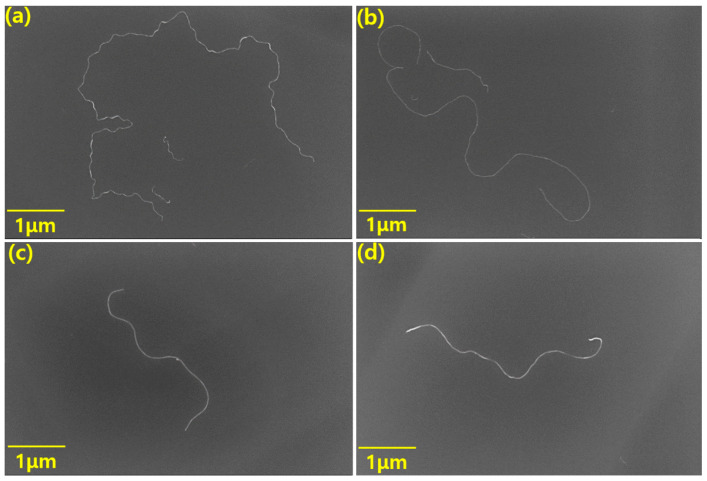
SEM images of MWCNTs: (**a**,**b**) in the case of high aspect ratio MWCNT (HCNT) with an average length of 12 μm and (**c**,**d**) in the case of low aspect ratio MWCNT (LCNT) with an average length of 4.5 μm.

Figure 2 shows the SEM images of the two kinds of MWCNTs. In order to obtain SEM images of a single MWCNT, we physically separated them from the bundles by ultra-sonication. We took enough SEM images to determine the average length of each MWCNT. From Figure 2a,b, we calculated the average length of the longer MWCNT, which was found to be 12 μm with an aspect ratio of 2400. The MWCNT was named the high aspect ratio MWCNT (HCNT). We measured the mean length of the shorter MWCNT in Figure 2c,d and determined it to be 4.5 μm with an aspect ratio of 900. This was named the low aspect ratio MWCNT (LCNT). Furthermore, we found that HCNT is more twisted than LCNT.

**Figure 3:**
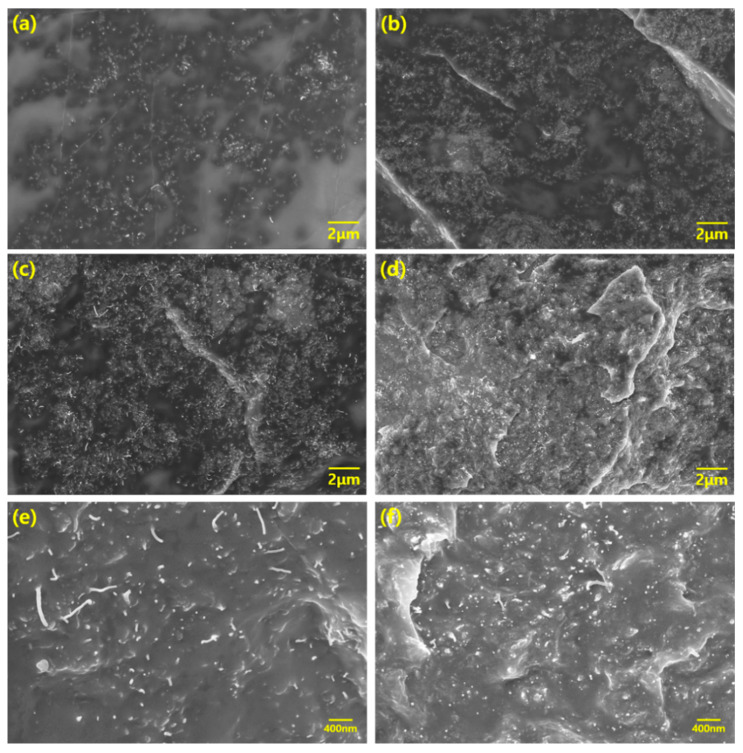
SEM images of MWCNT/PDMS composites: (**a**) 1 wt% LCNT/PDMS, (**b**) 1 wt% HCNT/PDMS, (**c**) 5 wt% LCNT/PDMS, (**d**) 5 wt% HCNT/PDMS with low resolution, and (**e**) 5 wt% LCNT/PDMS, (**f**) 5 wt% HCNT/PDMS with high resolution.

We obtained the SEM images to confirm that the MWCNTs were uniformly dispersed on the composites by three-roll milling and how the internal morphology of the composite was constructed according to the aspect ratios and content of MWCNTs. Figure 3 shows SEM images of morphologies of the 1 and 5 wt% LCNT/PDMS and HCNT/PDMS composites, respectively. In Figure 3a,b, we observe that both LCNT and HCNT were uniformly dispersed on the composites.

We discerned that high contents of both LCNT/PDMS and HCNT/PDMS are also evenly dispersed in Figure 3c,d. From Figure 3e,f, we observe that the fillers were more densely packed into the HCNT/PDMS composite than into the LCNT/PDMS composite at the same PDMS content. This difference in the density of filler packing is attributed to the longer length of HCNT relative to that of LCNT, which makes HCNT observation more likely. 

### 3.2. Mechanical Properties

**Figure 4:**
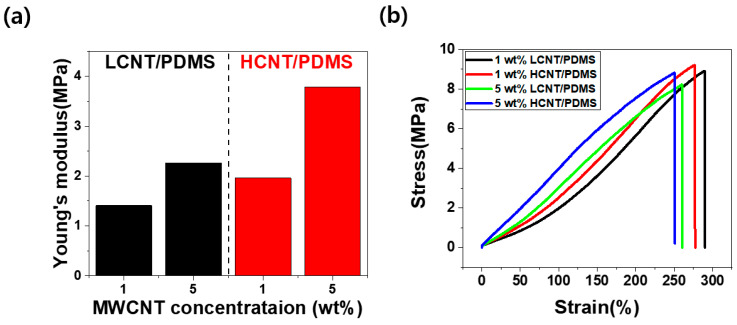
Mechanical properties of MWCNT/PDMS composites: (**a**) Young’s modulus of LCNT/PDMS composites (black bar) and HCNT/PDMS composites (red bar), (**b**) strain–stress curve of the 1 and 5 wt% LCNT/PDMS and HCNT/PDMS composites.

Tensile tests were conducted to find out the mechanical properties according to the aspect ratio and content of MWCNTs. Figure 4 shows the mechanical properties for each composite. We obtained Young’s modulus(Elastic modulus) using Hooke’s law, as given by

σ = εE(1)

where σ is the stress, ε is the strain, and E is Young’s modulus. In Figure 4a, we observed that Young's modulus increased as the filler content and aspect ratio increased. This finding means that composites are more mechanically reinforced at higher aspect ratios and higher MWCNT contents. The reason for this is that the MWCNTs tend to bridge the polymer cracks. In this respect, HCNT is more advantageous than LCNT due to its greater length. Furthermore, load transfer from polymer to MWCNT occurs, mechanically reinforcing the composites. The longer length of HCNT also makes it more advantageous for load transfer compared to LCNT. The bridge effect and the load transfer of CNT were confirmed in previous papers [23,24,25]. Moreover, assuming that the filler is uniformly dispersed, increased filler content naturally increases the mechanical reinforcement of composites because of the increased load transfer and polymer bridge effects mentioned above.

However, Figure 4b shows that tensile elongation at break (ε_break_) decreased with a higher aspect ratio of MWCNTs and larger content of MWCNTs. In the 1 wt% composites, ε_break_ decreased from 290.22% (LCNT/PDMS) to 277.48% (HCNT/PDMS) with an increasing aspect ratio of MWCNTs. In the 5 wt% composites, ε_break_ decreased from 260.65% (LCNT/PDMS) to 251.05% (HCNT/PDMS) with an increasing aspect ratio of MWCNTs. Furthermore, as the filler content increased, the tensile stress decreased slightly. These phenomena were caused by a shortage of interfacial interplay between the MWCNTs and PDMS. Namely, MWCNTs are likely to exist in the bundle state or in aggregated form at high content, hence resulting in the poor interaction between MWCNT and PDMS. Therefore, the tensile stress decreased slightly, as shown in Figure 4b. As the filler content increased, the reduction of tensile stress and the decrease in the interaction between filler and polymer were also observed in previous studies [26].

### 3.3. Electrical Conductivity and Percolation Threshold

**Figure 5:**
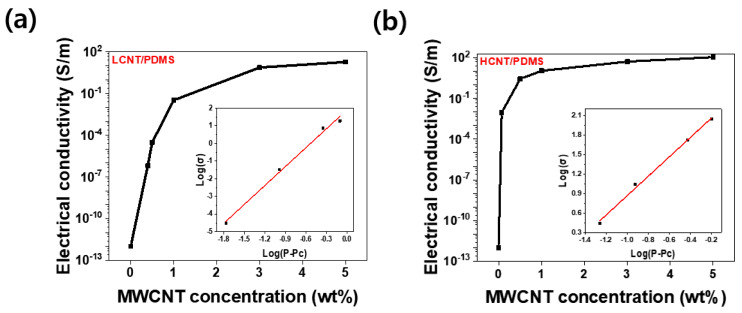
The electrical conductivity of MWCNT/PDMS composites as a function of MWCNT wt% and electrical percolation threshold of MWCNT/PDMS composites. Inset: log-log plot of the conductivity of composites according to the relation (P−P_c_): (**a**) LCNT/PDMS composites case and (**b**) HCNT/PDMS composites case.

The electrical properties of the composite are mainly determined by the aspect ratio and content of the filler. Figure 5 shows the variation in the electrical conductivity of LCNT/PDMS and HCNT/PDMS with content. The electrical conductivity (σ_electrical_) is calculated by Equation (2).

(2) $$\sigma_{\mathrm{electrical}}=\;\frac{\ell }{\mathrm{SR}}$$

In Equation (2), ℓ is the length between the electrodes, S is the cross-section of the sample, and R is the electrical resistance of the sample. We observed that at the same content, HCNT/PDMS has a higher electrical conductivity than LCNT/PDMS. This is because HCNT has a greater length, making it advantageous for the formation of conductive paths inside the composites. Previous papers also showed that fillers with higher aspect ratios have larger conductivity at the same content [14,16]. 

Furthermore, sharp increases in electrical conductivity were observed at 0.4 wt% for LCNT/PDMS and at 0.07 wt% for HCNT/PDMS. This phenomenon was explained by the percolation theory [27,28]. The power law for P > P_c_ is shown in Equation (3) below, where σ is the electrical conductivity of the composites, σ_z_ is the reference electrical conductivity, P is filler content, P_c_ is percolation threshold, and t is a critical exponent.

σ = σ_z_(P − P_c_)^t^ (3)

From Equation (3), considering σ_z_ as a constant value, we obtained that Pc = 0.4 wt% and t = 3.58 for LCNT/PDMS, whereas P_c_ = 0.07 wt% and t = 1.49 for HCNT/PDMS. Calculated values of P_c_ and t for the LCNT/PDMS and HCNT/PDMS composites followed the trends of the same values observed in previous studies [16,26,29,30,31]. We observed that HCNT/PDMS had a lower percolation threshold than LCNT/PDMS. HCNT has the advantage of the formation of conductive paths as it is longer than LCNT. The advantage comes in that even at lower filler contents, conductive paths are effectively formed; hence, HCNT/PDMS has a lower percolation threshold than LCNT/PDMS. Furthermore, electrical conductivity tended to be more saturated as the filler content increased. This is because enough conductive paths were already formed on the composites above a certain filler content, so that the addition of more filler results in only weak improvements in the electrical conductivity.

### 3.4. Piezoresistive Properties in Cyclic Loading

**Figure 6:**
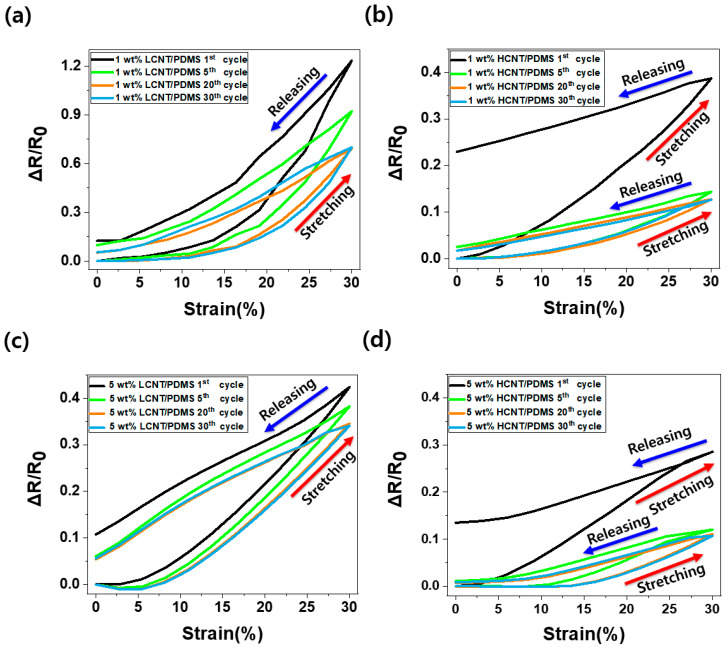
Normalized change in resistance (ΔR/R_0_) versus strain for MWCNTs/PDMS composite under discontinuous cyclic tensile deformation: (**a**) The case of 1 wt% LCNT/PDMS composite, (**b**) the case of 1 wt% HCNT/PDMS composite, (**c**) the case of 5 wt% LCNT/PDMS composite, and (**d**) the case of 5 wt% HCNT/PDMS composite.

Figure 6 shows the ΔR/R_0_ (relative change in resistance) of the sample under tensile deformation (where R is the real-time resistance of the samples, R_0_ is the initial resistance of the samples, and ΔR is R−R_0_). In Figure 6, when the 1 wt% and 5 wt% LCNT/PDMS and HCNT/PDMS composite samples were stretched to 30% strain, the relative change in resistance decreased from 123% to 70%, from 39% to 13%, from 42% to 34%, and from 29% to 11%, respectively, as the number of cycles increased, and the relative change in resistance was finally saturated after more than 20 discontinuous cycles. The hysteresis values of 1 wt% and 5 wt% of LCNT/PDMS and HCNT/PDMS also reduced from 12% to 5%, from 23% to 2%, from 11% to 6%, and from 14% to 8%, respectively, and they were then eventually saturated as well. 

Here, we observed the decrease of relative change in resistance following the repeated cycles. Moreover, the values of relative change in resistance and hysteresis were eventually saturated when the number of cycles exceeded 20. This is because the morphology of MWCNTs inside the composites is rearranged with repeated constant stretching and releasing. The relative change in resistance and hysteresis value changed depending on the morphology of the filler, and the change in the morphology of the fillers was saturated under repeated constant strain and releasing cycles. Therefore, relative change in resistance and the hysteresis value were stabilized depending on the stabilization of the filler morphology.

Comparing the relative change in resistance of LCNT/PDMS and HCNT/PDMS, we observed that LCNT/PDMS showed a better piezoresistive effect and stable hysteresis properties after the 1st cycle. The reason for the better piezoresistive properties of LCNT/PDMS is that LCNT is shorter than HCNT, so that the connections between LCNTs are easily broken during tension. Furthermore, the wavy HCNT expanded during tension, thus exhibiting an advantage by maintaining contacts between the HCNTs. According to previous literature, the expansion of the wavy HCNT negatively affects its piezoresistive effect [32,33]. Therefore, a better piezoresistive effect is observed in LCNT/PDMS. In terms of hysteresis properties, LCNT/PDMS shows smaller hysteresis properties than HCNT/PDMS because LCNT has a lower tendency for buckling than HCNT upon release from tensile deformation. Theoretical analyses presented in previous papers confirm this phenomenon [20]. In other words, the electrical paths inside the LCNT/PDMS are less restructured than the electrical paths inside the HCNT/PDMS when released after stretching.

**Figure 7:**
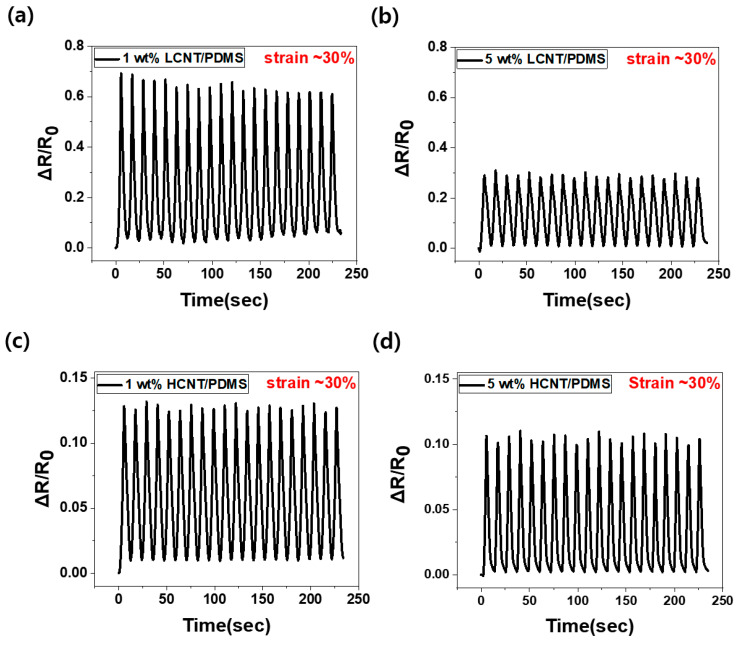
Normalized change in resistance (ΔR/R_0_) under 20 cycles of continuous tensile deformations to 30% strain: (**a**) Case of 1 wt% LCNT/PDMS, (**b**) case of 5 wt% LCNT/PDMS, (**c**) case of 1 wt% HCNT/PDMS, and (**d**) case of 5 wt% HCNT/PDMS. (For all samples, 30 pre-discontinuous cycles were executed before the continuous cyclic test).

Figure 7 shows the relative changes in resistance according to the aspect ratio and filler contents for 20 cycles of continuous stretching and releasing. The part where the relative change in resistance increases is the stretching state (up to ~30% strain), and the part where the relative change in resistance decreases is the releasing state. We observed that the relative change in resistance increased as the filler content decreased. This is because the contacts between the MWCNTs inside the composites are more likely to break during stretching at low filler content.

When MWCNTs of low filler content composites are rearranged during tensile stretching, new contacts are less likely to occur than in composites with higher filler contents. Namely, the relative change in resistance is large in the composites with low filler content because the morphology of the MWCNTs changes due to the tension, and there is more contact loss than formation of new contacts. Furthermore, according to the antecedent papers, contact loss results in increasing tunneling resistance and has a positive effect on the piezoresistive effect [34,35].

As the filler content increased, the relative change in resistance in the LCNT/PDMS composite decreased from 69% to 29%, a decrease of approximately 58%, whereas the relative change in resistance in the HCNT/PDMS composite decreased from 13% to 11%, a change of approximately 2%. The difference in the relative change in resistance between the 1 wt% and the 5 wt% LCNT/PDMS is greater than that in HCNT/PDMS. As we saw above, HCNT/PDMS has a low percolation threshold (at 0.07 wt%), so sufficient conductive paths exist even at 1 wt% composite, whereas LCNT/PDMS has a large percolation threshold (at 0.4 wt%), and it results in the insufficiency of the conductive paths at the same wt% composite. Therefore, the increased content, from 1 to 5 wt%, would have a great effect on the conductive paths in the LCNT/PDMS. On the other hand, the conductive paths in the HCNT/PDMS were not significantly affected by the increment. Moreover, we observed stable and constant relative change in resistance over 20 cycles of continuous stretching and releasing. 

## 4. Conclusions

To investigate the strain-sensing properties according to the aspect ratio and content of MWCNTs within composites, 0.4, 0.5, 1, 3, and 5 wt% LCNT/PDMS and 0.07, 0.5, 1, 3, and 5 wt% HCNT/PDMS composites were fabricated. From the tensile tests, we confirmed that using low filler content with LCNT resulted in a lower Young's modulus and a larger strain range than using high filler content with HCNT due to the bridge effect of the filler and loading transfer. In addition, by measuring the electrical conductivity, we proved that LCNT/PDMS had a lower electrical conductivity and a larger percolation threshold than HCNT/PDMS because of the poor ability of LCNT to create electrical paths in the composites. However, through the discontinuous cycle tests, we confirmed that LCNT/PDMS exhibited a more sensitive piezoresistive effect and stable hysteresis properties than HCNT/PDMS because LCNT has a short length while HCNT is characterized by waviness. Furthermore, piezoresistive effect and hysteresis value were saturated according to the repeated constant strain and releasing. Therefore, for the stable use of the strain sensor, pre-stretching is required in a larger range of strains than the strain section we will apply. Finally, through continuous cycle tests, we found that low filler content resulted in a stronger piezoresistive effect than high filler content since there is greater total conductive path loss at lower filler content than at higher filler content composite during stretching. In conclusion, composites with lower aspect ratio and smaller filler content are great condition in terms of piezoresistive effect and strain hysteresis.
